# Unlocking the Power of Exosomes for Crossing Biological Barriers in Drug Delivery

**DOI:** 10.3390/pharmaceutics13010122

**Published:** 2021-01-19

**Authors:** Rebekah Omarkhail Elliott, Mei He

**Affiliations:** 1 Department of Chemical and Petroleum Engineering, Bioengineering Program, University of Kansas, Lawrence, KS 66045, USA; relliott@gus.pittstate.edu; 2 Department of Chemistry, University of Kansas, Lawrence, KS 66045, USA; 3 Department of Pharmaceutics, College of Pharmacy, University of Florida, Gainesville, FL 32610, USA

**Keywords:** exosomes, biological barriers, drug delivery, tight junctions, precision medicine

## Abstract

Since the 2013 Nobel Prize was awarded for the discovery of vesicle trafficking, a subgroup of nanovesicles called exosomes has been driving the research field to a new regime for understanding cellular communication. This exosome-dominated traffic control system has increased understanding of many diseases, including cancer metastasis, diabetes, and HIV. In addition to the important diagnostic role, exosomes are particularly attractive for drug delivery, due to their distinctive properties in cellular information transfer and uptake. Compared to viral and non-viral synthetic systems, the natural, cell-derived exosomes exhibit intrinsic payload and bioavailability. Most importantly, exosomes easily cross biological barriers, obstacles that continue to challenge other drug delivery nanoparticle systems. Recent emerging studies have shown numerous critical roles of exosomes in many biological barriers, including the blood–brain barrier (BBB), blood–cerebrospinal fluid barrier (BCSFB), blood–lymph barrier (BlyB), blood–air barrier (BAB), stromal barrier (SB), blood–labyrinth barrier (BLaB), blood–retinal barrier (BRB), and placental barrier (PB), which opens exciting new possibilities for using exosomes as the delivery platform. However, the systematic reviews summarizing such discoveries are still limited. This review covers state-of-the-art exosome research on crossing several important biological barriers with a focus on the current, accepted models used to explain the mechanisms of barrier crossing, including tight junctions. The potential to design and engineer exosomes to enhance delivery efficacy, leading to future applications in precision medicine and immunotherapy, is discussed.

## 1. Introduction

**Figure 1:**
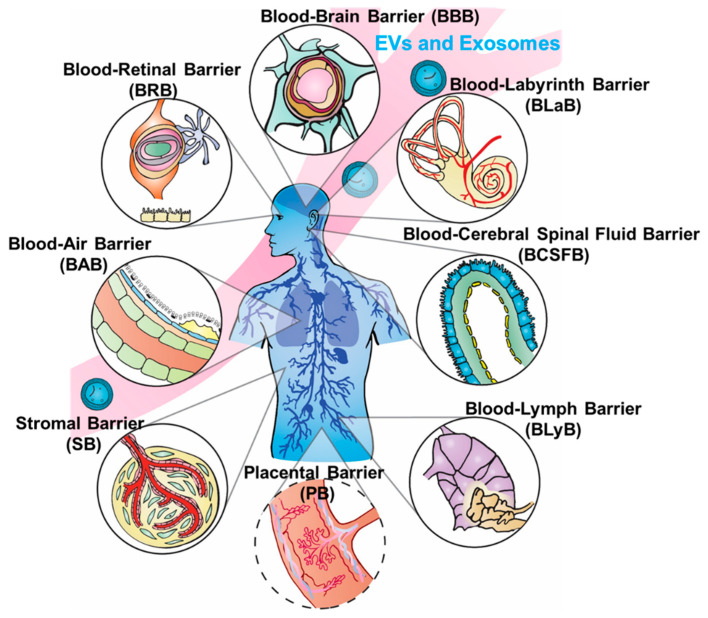
Illustration of essential biological barriers penetrated by exosomes for cellular regulation and delivery.

Biological barriers (BBs) confer protection defending against invading pathogens and disease, yet simultaneously complicate drug delivery to target sites. The human body contains numerous BBs surrounding specific tissue regions and organs, including the blood–brain barrier (BBB), blood–cerebrospinal fluid barrier (BCSFB), blood–lymph barrier (BlyB), blood–air barrier (BAB), stromal barrier (SB), blood–labyrinth barrier (BLaB), blood–retinal barrier (BRB), and placental barrier (PB) (as illustrated in Figure 1), and many others. We chose the most relevant barriers for potential development of exosome delivery platforms based on the significance and influence of barriers to each other, as well as the spatial order illustrated in Figure 1. Some BBs such as the BBB and BCSFB have overlapping similarities, yet each present distinct membranes, fluids, receptors, or mechanisms preventing therapeutic substances from crossing. Tissues in BBs can have different origins and unique fluids as observed in the perilymph versus cerebrospinal fluid (CSF). The innate function of BBs hinders drug delivery and uptake, precluding efficacious therapeutic interventions. Advances, such as in the treatment of cancer, auditory dysfunction, fetal morbidities, genetic disorders, and ocular diseases, continue to be hampered by unique physical structures and microenvironment in differing BBs [1]. On the other hand, inappropriate intervention methods can interrupt natural barrier function, in turn increasing potential risks of infection or open channels for pathogenicity [2]. Thus, a delicate balance is critical between protections conferred and restrictions defined by BBs that would permit access to therapeutic interventions without compromising the integrity of physical structures. BBs not only impede treatment options but diminish bioavailability of drugs in regions protected by BBs, which can lead to increased drug resistance in bacteria and other pathogens. Moreover, engineered nanoparticles and drug therapies for crossing BBs could potentially dysregulate or suppress biochemical pathways and increase risks of side effects [3,4,5,6].

Drug delivery approaches have focused on the pharmaceutical access to BB protected tissues. Dendrimers, nanoparticles, liposomes, hydrogels, and cell-penetrating peptides (CPPs) have each risen and fallen as hopeful candidates [7]. Therapeutic access has proven elusive, however, due to immunogenicity, non-specificity, and insufficient bioavailability in vivo. Modifications, such as hybrid and gold-enhanced nanoparticles, have been implemented to counter such issues [8]. Recently engineered products include nanogels (NGs), which have been designed to mimic endogenous particles [9]. Techniques to allow controlled release of cargo in response to pH, light, and enzyme activity have been utilized to increase penetration through BBs but remain problematic [10]. In the BB microenvironment, efflux pumps, tight junctions, molecule size for passive transport, as well as receptor-mediated transport and enzyme saturation are key factors in achieving effective uptake and bioavailability of drugs, which is critical for developing safe and effective delivery strategies.

Recently, extracellular vesicles (EVs) have been reported to provide much needed access with their intrinsic ability to penetrate BBs. Although the definition of EV subcategories (e.g., exosomes, microvesicles, and apoptotic vesicles) continues to be debated, the nanosized exosomes (30–150 nm) derived from the endosome pathway have drawn attention on their natural ability to cross BBs, due to their readily available targeting capabilities and endogenous, specific homing markers [11]. For example, studies demonstrate that exosomes contain receptors, such as transferrin, LDL, and insulin, which are known to allow uptake via the BBB [12]. A recent study in psychopathological conditions also showed that exosomes were internalized in microglial cells, which forms part of the neurovascular unit of the BBB. Furthermore, exosomes are amenable to surface modifications that improve inherent targeting and transport abilities. While transport properties of exosomes are not fully understood, strategic exploitation of advantageous properties of exosomes has led to several innovations that can increase delivery and uptake efficacy via increasing the penetration through various BBs [13,14].

As evidenced in this review, increasing data demonstrate that exosomes are ideal carriers for drug delivery, and exploitation of their properties could lead to more successful BBs penetration. The mechanisms by which exosomes cross biological barriers is still limited, but this knowledge is critically needed to enter a new paradigm for preventive and therapeutic interventions via drug delivery. This review covers state-of-the-art of exosome research on crossing several important biological barriers (illustrated in Figure 1) as detailed in the following subsections, with the focus on the current accepted models used to explain the mechanisms of barrier crossing. The potential to engineer exosomes to unlock the power of advanced delivery is discussed using current accepted models of barrier crossing mechanisms, which could lead to future applications in precision medicine and immunotherapy.

## 2. Blood–Brain Barrier (BBB) and Blood–Cerebrospinal Fluid Barrier (BCSFB)

The blood–brain barrier (BBB) is one of the most extensively studied barriers within the body yet one of the most formidable barriers to overcome. This is due to numerous complications involving the many factors needed to maintain the integrity and function of the BBB, including regulating cerebral blood flow, permeability and preservation such as the highly specialized endothelial cells within tight junctions [15,16], mobile pericytes, mitochondria [17,18,19,20,21,22,23,24], astrocytes, solute carriers [25], multidrug transporters, efflux pumps, ectozymes, and endozymes [15,25]. Additionally, the BCSFB plays an integral role in BB function in tandem with the BBB. It has a much larger role in neuropathology, neuromedical translation, and neurodegeneration [26], as seen in aging and Alzheimer’s Disease (AD) [26,27,28,29], and potentially brain tumor invasion [30], which is discussed together with the BBB. Both the BCSFB and BBB interfaces coordinate together, systematically regulating carrier transport while simultaneously allowing solute exchange. Though similar and working in unison, there are significant differences in function and composition of these two barriers. For example, while endothelial cells and tight junctions delimit the main boundary of the BBB and reduce permeability, the morphology of the BCSFB in contrast is more characterized by the leaky epithelia function of the choroid plexus providing much less resistance than the BBB, thereby maintaining more isotonic transepithelial concentration gradients [31]. The cohesive function of these distinctive yet similar barriers further impedes therapeutic progress. Understanding and exploiting BCSFB properties should continue to be explored in conjunction with the BBB and could provide more efficacious treatments involving central nervous system (CNS) disorders.

Recently, exosomes have been studied as a means of crossing the BBB and BCSFB [32,33], due to their natural homing specificity, immunogenicity [34], and prolonged half-life in the blood [35,36], heralding an endogenous approach to a complex puzzle. However, the role of exosomes in barrier integrity has still been largely unknown. Exosomes have been shown to carry cargo such as miR-3p across the BBB; alleviate neuroinflammation in hemorrhage regions in the brain [37]; and transport anticancer agents specifically targeting neurons [32], microglia, and oligodendrocytes [38]. They can also be uptaken by brain parenchyma and other cells in the brain [39]. Exosomal miRNAs and lncRNAs have been shown to inhibit uptake of glucose in brain astrocytes [40], mediating microglial cell polarization [41], but relatively few studies identify precise barrier mechanisms. One interesting study demonstrated that increased permeability of the BBB resulted in a greater influx of exosomes into brain microvascular endothelial cells (BMECs) [42,43] with migration through BMECs found primarily to be through the transcellular route rather than tight junctions involved in the paracellular pathway [42]. Exosomes also carry miR-132, which is established as a regulator of adherens-junction-related proteins, increasing BBB permeability and microhemorrhage events in brain microvasculature [44]. A recent study demonstrated that neural stem cell-derived exosomes can both initiate and repair BBB destruction, mitigating and reversing BBB-induced AD [45]. Such essential roles of exosomes in BBB integrity indicate an ever-increasing therapeutic value.

The size of nanoparticles was initially recognized as one of the key criteria for crossing BBs. In contrast, exosome origin, cargo load, and surface proteins exhibit greater influence and have been explored, not only leading to better characterization but also to improved understanding of exosomal uptake mechanisms in crossing BBs. It has been shown that factors other than size are key in exosome transport. The origin of exosomes derived from endothelial brain cells could affect drug delivery capability [32]. In preliminary studies, despite all four exosomes being derived from brain endothelial cells and displaying cell internalization, only the comparatively larger bEND.3 exosome was able to successfully cross the BBB and thereby deliver anti-cancer agents effectively, due possibly to a higher presence of tetraspanin surface protein CD63 [32]. Therefore, more research efforts are needed to consider surface modification and engineering to enhance the exosome delivery ability. For example, exosomes became concentrated in the brain after surface modification with overexpression of the rabies virus glycoprotein (RVG) peptide [38]. Ye et al.’s functionalized, methotrexate-loaded exosomes with LDL peptide demonstrated increased exosome extravasation of the BBB [46]. Grapp et al. utilized exosomes containing folate receptor-alpha (FOLR1) to demonstrate BBB access via the BCSFB [47]. In another study, mesenchymal stromal cell-derived exosomes loaded with miR-210 demonstrated improved targeting of ischemic brain when conjugated with c(RGDyK) peptide, indicating increased angiogenesis and resulting in a significant increase in animal survival [48]. The reported studies indicate that exosomes enable enhanced penetration of the BBB through means other than size and permeability, which sheds light on induction and modulation of brain diseases.

## 3. Blood–Lymph Barrier (BLyB)

The functional linkage between the BBB and the blood–lymph barrier (BLyB) is currently being explored and unraveled [49,50]. New studies observed circulation of both CNS-derived molecules and lymphocytes within the brain as well as drainage of CSF into cervical lymph nodes via glymphatic system [51,52]. These findings present the interesting possibility of surmounting the BLyB in order to gain access to the brain parenchyma and perhaps bypass other biological barriers within the body. Rapidly growing evidence in the past two decades proves lymphatic system interactions in the CSF and brain via the glymphatic system [53], which may be the key to both pathological invasions and treatment, as well as maintaining biological function and homeostasis [54]. However, challenges in devising effective drug delivery in such a dynamic system are daunting.

The blood–lymph barrier is physiologically a complex, heterogenic lymph endothelium barrier [55,56], most commonly considered as the blood–thymus barrier. Factors that control passing the BLyB include problematic extravasation, penetration to the interstitium, fast or slow diffusion, and mucosa [55]. Beyond these basic components, the reticular network (RN) made up of collagen fibers restricts entry of soluble materials, which creates lymphocyte microenvironments [57,58,59,60,61,62]. Adding to the complexity of this system, the RN connects the subcapsular sinus to high endothelial venules (HEVs) [63] permitting movement through the cortex, but is itself sheathed in fibroblastic reticular cells creating a separate extracellular space from surrounding lymphocytes [64]. The BLyB is perhaps one of the most challenging BBs to cross and exploit, due to both the innate fluidity of the system as well as its complex composition. Many components, mechanisms, and functions are still unknown. Thus, drug delivery targeting the lymphatic system is more challenging than other BBs. Similar to how cancer cells exploit the lymphatic system as a channel for metastases to different regions in the body, chemotherapeutics packaged via nanoparticles have been explored as a means to selectively deliver agents to solid tumors intralymphatically and locally. T-cells, [65,66], monocytes [67], and the more specialized macrophages [68] are being considered as delivery platforms, with additional benefits such as the ability to easily cross the BLyB to provide controlled delivery and protect cargo from degradation [69]. The entrance into the BLyB can be easily identified; however, limited bioavailability is problematic due to immunogenicity and immune responses [70,71,72,73].

Employing exosomes to bypass the copious obstacles presented both within and without the BLyB is promising. Exosomes are the optimal size [74] and composition, being lipids rather than soluble [75], permitting passage through and travel within the BLyB including the RN. Exosomes may also offer systemic understanding of the BLyB’s effects in connecting multiple BBs, which are yet to be explored. Dendritic cell derived exosomes (DC-Exos) are known to be antigen presenting [76], and have been used to regulate the inflammatory response in arthritis [77] and stimulate T-cell lymphocytes [78]. A myriad of exosomal nucleic acids could also serve as biomarkers for therapy resistance as observed by upregulated exosomal miRNAs in diffusive large B-cell lymphoma [79] and Hodgkin lymphoma [80]. Using in vivo near-infrared imaging, Srinivasan et al. [74] observed exosome delivery from periphery to lymph nodes (LNs) within five minutes, with steady state in the nodes achieved within 30 min. Additionally, exosomes were present for up to two days in the LNs and had also collected in other organs [74].

Through regulation in the lymphatic system with targeted exosomal drug delivery, researchers can design potent tools for treating cancer and other diseases connected with the lymphatic system. Exosomes derived from lymphatic endothelial cells (eLECs) have been explored for lymphatic transportation and immunity mediation [81,82] in studies such as those of Srinivasan et al., 2016, who demonstrated the lymph system’s key role in transporting exosomes, and Brown et al., who showed that exosome-rich endothelial vesicle (EEV) fractions are released by human LECs upon exposure to inflammatory cytokine TNFα [74,83]. DC-Exos have been seen to induce immune responses through DCs and T-cell stimulation of cytokine release with the potential significance of membrane components such as increased C-C chemokine receptor 7 (CCR7) correlating to an increased response in lymphoid organs [84]. More understanding of recipient cells, transit, surface ligands, and cargo of exosomes, along with their access to the lymphatic system and movement across and within the BLyB, is needed for the development of a facile therapeutic pathway. An increasing amount of research on engineered DC exosomes, including surface receptor engineering [85] and cargo loading [86], has been conducted for more potent and targeted delivery in developing cancer immunotherapy [87,88,89,90] and other disease treatments [91]. Knowledge of exosome transport mechanisms within the BLyB could potentially simplify the complexity of designing cell-specific therapies or strategies and herald rapid, personalized medical care for patients.

## 4. Blood–Air Barrier (BAB)

The BAB plays a key role in effectively restricting movement of pathogens, but this defense mechanism also inhibits therapeutic interventions [92]. Obstruction and clearance of pathogens as well as therapeutic drugs is accomplished through three main barriers: mechanical, chemical, and immunological barriers [93]. Mucosa, composed of the epithelium, lamina propria, and smooth muscle, also contains numerous immunological cells including neutrophils, macrophages, T and B lymphocytes, defensins, chemokines, and cytokines [94]. The complex network of the “bronchial tree” in the alveolated region [95] protects the epithelial layer in the pulmonary air space. Alveolar epithelium is where gas exchange and surfactant secretion occur [96,97]. Microbicidal effector molecules, epithelial tight junctions with potentially targetable signaling molecules, and lung epithelial cytosolic pattern recognition receptors (PRRs) are new areas of exploration that also guard and monitor the BAB [98].

Some materials bypass the mucus layer and are deposited on the lung surface, but there they undergo surveillance and expulsion. Harmful substances or drug therapies can be cleared from the lungs through the ciliary action of a periciliary liquid layer underneath the lung mucus [99]. Mucociliary clearance involves moving substances from the conduction airways to the oropharynx to be swallowed, ingested, or expectorated [99,100]. Inhaled therapeutics face an additional challenge, competing with this mechanical barrier as complete clearance of the conducting (tracheobronchial) airways occurs within 24 h [95]. This clearance is also aided by macrophage clearance [99,101]. Furthermore, substances can be chemically inactivated through proteolytic enzymes such as neutral endopeptidase and cathepsin H [92,102].

Literature on the role of exosomes within the BAB system mainly focuses on disease pathogenesis such as asthma and immunological responses, but an understanding of exosomal crossing of the BAB to deep lung tissue is still lacking. Exosomes express MHC I and II [103,104], and interact with professional immune cells [105] in the aerodigestive barriers to regulate mucociliary clearance [106]. Thus, reprogramming or utilizing exosomes to regulate protective mechanisms within the BAB could provide potentially powerful tools for working within the BAB rather than trying to surmount it. Another interesting avenue of exploration involves exosomes in lung injury and aging lungs for remodeling, which has not been well explored but would be worthwhile, especially in light of the COVID-19 pandemic. Research also demonstrated that exosomes maintain chronic inflammation within the nasal cavity [106], induce pro-inflammatory conditions in bronchial epithelial cells [107,108,109], and play a role in pro-inflammatory conditions in pulmonary diseases. Additionally, the dialogue between cytokines and exosomes is fascinating. Exosomes showed a promising role in mounting immune defenses by activating macrophages and recruiting neutrophils [110], which is critical in eliminating pathogens. Significantly, exosomes appear to play a role in both the innate and adaptive immune system within the BAB [107] and may even play a key role in chronic lung allograft dysfunction [111]. On the other hand, exosomes have also been observed to contribute to excess mucus secretion and tissue destruction [112]. Thus, the means to reengineer exosomes to undo deleterious effects is potentially powerful, but caution is vital. There is still much to be understood not only about the BAB but in regard to exosomes and their roles in pulmonary heath and disease, which could provide new delivery options and novel therapies.

## 5. Stromal Barriers (SBs)

Stromal barriers (SBs) are the only pathological barrier presented in this review, proving to be formidable obstacles in cancer therapy. Cancer remains one of the leading causes of death globally. Despite increasing efforts and novel therapeutics, solid tumors prove to be refractory to promising treatments including nanodrugs, antibodies, small molecule drugs, and antibody drug conjugates [113,114,115,116,117,118,119,120,121]. Furthermore, innate and acquired drug resistance [122], insufficient specific penetration, and limited therapeutic target identification contribute to subpar outcomes [123,124]. Upon access to malignant tissue, as observed in epithelial-derived cancers for example, heterogeneity of the SBs in the same and different anatomic locations [125] makes the targeted delivery more complicated. Crosstalk between stromal cells and cells within the tumor microenvironment (TME) [126] presents multiple obstacles as well [127,128,129]. Fibroblasts within the TME communicate with tumor cells, which can increase refractory tumors [130]. In addition to these challenges, all systemically delivered molecular therapeutics utilize transendothelial delivery, which requires high drug concentrations for passive passage across tumor barriers, which in turn leads to drug toxicity and reduced feasible usage across multiple drug classes [131,132,133,134,135,136,137,138,139,140,141,142,143,144,145,146,147]. For instance, less than 0.1 to 1 percent of drug accumulates in tumors from fairly high drug doses [131,132,133,134,135,136,137,138,148,149], which leads to a tumor barrier causing treatment failure. SBs are increasingly significant in patient outcome, resistant malignancies [113,150,151,152,153], and invasiveness [150,151]. Within the stroma itself, substantial heterogeneity of cancer-associated fibroblasts (CAFs) not only impedes cancer therapies [154], but also leads to various responses by the SB in regard to inflammatory responses and potential correlation to malignancy refractory [127,155,156,157,158,159,160,161]. These and other findings have made SBs attractive targets for cancer therapies; however, conflicting outcomes demonstrate the need for further investigation.

Crosstalk within the TME brings up many intriguing questions regarding the role of exosomes and their dialogue within this complex environment [162,163]. Exosomes within the TME have already proven to be key players in response to hypoxia, oxidative stress, and acidosis triggering remodeling of the TME and a stromal response leading to malignancy [125,164]. Exosomes regulate pathways that increase therapeutic resistance [165], yet over the several years of research connecting exosomes to enhanced tumor resistance and invasiveness, only in the past two to three years has the TME and exosome connection begun to be more fully explored. To date, exosomes have primarily been studied to define their roles in carcinomas within the TME in regard to being secreted by cancer or stromal cells [165,166,167]. In light of this substantial role, exosomes have demonstrated the next step in cancer treatment, which will involve using exosomes as carriers to interrupt tumor signaling or their involvement in restructuring the TME for inducing healthy tissue growth. Due to the complex signaling involved with exosomes, a database dedicated solely to their role in the tumor–stroma, extracellular matrix (ECM), CAFs, and different pathways would be strongly advised as a direct reference for scientists to continue building on previously established information. Some studies are already demonstrating various cargos carried by exosomes leading to apoptosis of immune cells [168,169,170,171,172], initiation and stimulation of cancer growth [173], and regulation of the tumor microenvironment (TME) [174]. More studies are still needed to fully exploit the advantages of exosome communication and effectively utilize exosomes to bypass the complex SB system.

## 6. Blood–Labyrinth Barrier (BLaB) and Blood–Retinal Barrier (BRB)

Because of the similarity in nature between the BlaB and BRB, these two barriers are discussed in one section to illuminate the current extent of research and relevant exosome therapy development. The stria vascularis and spiral ligament were defined as integral structures for the BLaB barrier [175] recognized by Hawkins in 1960. More specific structures within the stria vascularis were defined, such as tight junctions of marginal and basal cells within the endolymph and perilymph barriers, respectively [176]. Recently, a subset of barriers within the BLaB has been proposed, consisting of five interrelated but independent membranous labyrinth barriers: the blood–endolymph barrier, blood–perilymph barrier, cerebrospinal-fluid–perilymph barrier, middle-ear–labyrinth barrier, and endolymph–perilymph barrier [177]. These distinct, multiple barriers are enclosed within the otic capsule (bony or osseus labyrinth) with osseus and membranous layers presenting a formidable blockade. Each compartment requires different strategies to surmount these anatomical and physiological barriers as unique environments both permit and prevent barrier-specific transport.

Unique penetration for each barrier can be exploited for drug administration; however, the penetration also can conversely present inimitable challenges. Drug therapies have themselves been shown to cause damage. Ototoxic drugs, such as kanamycin, adriamycin, vincristine, and styrene, can affect cochlear barrier function, endolymphatic function, and outer hair cells of the cochlea, respectively [178,179]. Inflammation is also present with drug-related hearing loss [180,181,182]. As recently as 2018, exosomes had still not been explored or reported as being present in the inner ear. Since then, more and more researchers have begun to actively pursue exosome-based drug delivery instead of using synthetic nanoparticles (NPs), which could reduce potential side effects and enhance homing abilities. Furthermore, progress with synthetic NPs has been impeded with phagocytosis, opsonization [183], and aggregation [184]. In contrast, exosomes evade phagocytosis and macrophage degradation, and their extended circulation time within the body also allows for maximal drug delivery and uptake.

Interestingly, the BLaB contains components similar to the blood–retinal barrier (BRB) as well as the BBB. However, the limited studies relating to exosomes and ocular structures signify that much research is still needed in this area. With blood vessels comparable to both the BBB and BLaB, the retinal vascular epithelium (RVE), composed of tight junctions, forms one layer of the BRB. The retinal pigment epithelium (RPE) forms a second layer of the BRB, increasing complexity of access to interior regions. Unique barrier properties impact subretinal delivery as the RPE layer not only controls fluid absorption, but cells within this layer are among the most active phagocytes found in the body. Exosome binding or secretion of regulatory substances through ECM at barrier boundaries such as the RPE has shed light on diseases such as glaucoma and may eventually offer methods for improved therapeutic interventions [185,186]. Exosomes have been shown to potentially contribute to various impairment or reduction of function as seen in age-related macular degeneration (AMD) as they contribute to regulation of pigment granule formation and lipid homeostasis in RPE cells [187]. Exosomal microRNA affects tight junctions by downregulation of miR-105, which leads to vascular permeability and has been shown to impair the structural integrity of barriers [188]. Further research is called for particularly in these areas with therapies utilizing exosome carriers capable of microRNA and siRNA regulation. As an alternative to engineered NPs, exosomes could provide endogenous packaging, transport, and delivery, avoiding potentially toxic substances and substituting for viral vectors frequently used in gene therapies.

## 7. Placental Barrier (PB)

Barrier systems have some similarities such as the endothelium composing the anatomic substrate for the BB and inner BRB [189]. The placental barrier (BPB) likewise shares similarities with the blood–thymus barrier and outer BRB with the barrier composed of epithelial cells containing dense intercellular junctions, limited pinocytotic vesicles, and high expression of transporters increasing selectivity for exchange of molecules [189]. The PB, however, is unique among all barriers. The function is two-fold in that it protects the developing fetus from noxious elements while also serving as the key maternofetal interface [190,191]. Furthermore, the PB also functions as an endocrine organ [192,193,194]. Thalidomide-induced birth defects of the 1950s and 1960s altered the conception of the placenta as a drug impermeable barrier [192]. The chorionic villi, consisting mainly of cytotrophoblasts during the majority of pregnancy, form the main structure [195]. The placenta can be further divided into two main layers consisting of (i) a vascular network composed of syncytiotrophoblasts (STBs) in direct contact with maternal blood and (ii) trophoblasts [195,196,197] composing the outer layer with cytotrophoblasts functioning as stem cells for STBs, which re-epithelize injured or damaged sites [195,198,199]. Additionally, the STB layer functions as an endocrine organ synthesizing progesterone, estrogen, and growth hormones [200] with fetal endocrine signals from the placenta activating immune cells [201,202], blocking progesterone [203,204], and disrupting homeostasis in an inflammatory process readying the uterus for parturition [205,206]. Communication across the PB is vital for a healthy pregnancy with the placenta controlling and regulating this crosstalk [207]. However, maternofetal dialogue, such as in fetal metabolism across the PB, continues to challenge scientists and is still poorly understood for several reasons [197]. Unlike other barrier structures, the PB undergoes dynamic morphological changes throughout pregnancy to meet the demands of the developing fetus, in addition to altering the metabolism of the mother [208]. Furthermore, human placental tissue for studies is ethically limited [209], and animal models also differ significantly from human placenta [197,210]. The effective models for the trophoblast barrier have been lacking as cell line models lack vasculature and connective tissue [211]. The primary trophoblast cell lines that are self-renewing for long-term propagation have only recently become available for more thorough molecular studies [212,213]. The recent placental barrier-on-a-chip device could help to elucidate barrier crossing efficacy and replace animal studies or investigations, the results of which cannot be easily extrapolated to humans [214,215].

Drugs to treat disease can adversely affect both the mother and the developing fetus and may not be successful in crossing the PB. Therefore, more understanding of this incomplete barrier is necessary for effective and safe therapeutic interventions. Recently, researchers observed that lipopolysaccharide (LPS) stimulated macrophage-exosomes can prime immune effect on recipient cells [216] making them ideal candidates as inflammatory mediators in both fetal and maternal gestational tissues [217,218,219]. Newer studies are demonstrating that placenta-derived exosomes (PdEs) go beyond interventions for the mother and fetus, providing potential therapy for Duchenne muscular dystrophy [220] and spina bifida [221] as well as numerous applications for pregnancy disorders including gestational diabetes [222], monitoring fetal growth [223], in vitro fertilization (IVF) [224], and establishing and maintaining pregnancy [225]. Four main functions of placental exosomes have been studied in normal pregnancies: (i) maternofetal crosstalk [226]; (ii) maternofetal metabolic homeostasis [227]; (iii) maternofetal immune tolerance [228]; and (iv) regulation of angiogenesis and cell migration [226,227,229,230,231,232,233,234,235,236].

Being endogenous carriers, exosomes have tremendous potential to be utilized as transportation across the PB with their innate ability to not only to easily cross that barrier but also due to their numerous roles in pathogenesis and the immune system. Moreover, placental health could be monitored through exosomes from placental tissue such as STBs, which directly impact fetal progression and maternal modifications [237]. Furthermore, exosomal cargo and concentration changes throughout pregnancy [235,238] can be monitored to reflect stress, death, and activation of placenta for more efficacious interventions and therapies [239]. Overall, exosomes could provide insight into numerous unanswered questions and expand knowledge and treatment interventions for this challenging, dynamic barrier.

## 8. Discussion

The diversity and complexity of multiple, unique BBs have challenged and limited progress in both understanding and treating disease. While background and current progress are discussed here, this is not an exhaustive review as each barrier is fraught with numerous defenses, some of which are still unknown. For example, drug delivery at local sites must often cross BBs with mucosal surfaces, such as those seen in the BAB and BRB. Human mucus, however, is 10 to 100 micrometers thick on average and is intertwined with biomacromolecules and mucin in crosslinked fibers reinforced with disulfide bonds [240]. Although composed of 90 percent water, mucus has 10^3^ to 10^4^ times higher viscosity than water, which makes penetration much more challenging [241,242]. This is only one example of the copious obstacles therapies must overcome to bypass BBs.

**Figure 2:**
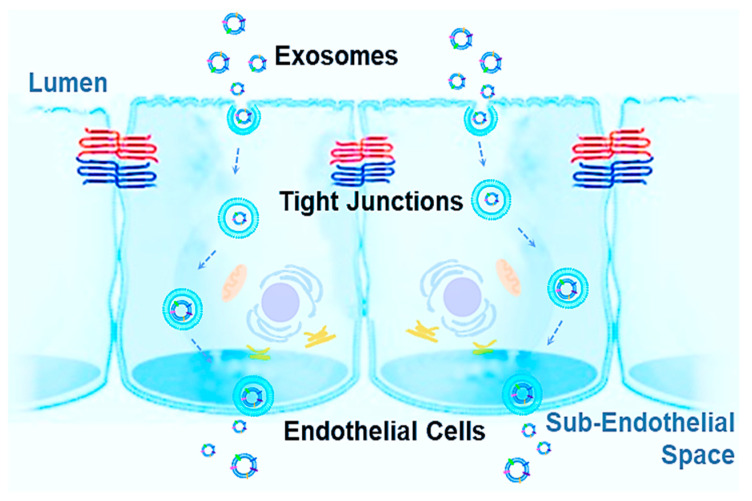
The schematic illustration of exosome transcytosis across biological barriers.

Additionally, increasing drug concentrations within tissues and systemically can not only contribute to resistance, as biodistribution and bioavailability impede efficacy, but risks and toxicity increase as well. Therefore, targeted therapies are needed with maximum drug dose absorption at the site of infection or disease. Successful treatment options, such as recent therapies like NGs, have been discovered in exploiting the dynamic properties employed by living organisms to respond to a constantly changing environment both within and outside the body [243,244]. For this reason, biological tools offer practicality in combatting unique biological defenses within the human body, especially important in BBs. Exosomes are uniquely positioned for tackling such challenges. Exosomes are innate vesicles, found in nearly every cell of the human body. As a result, the past two decades have seen tremendous growth in understanding exosomes, including both function, mechanisms, and compositions. There is a special emphasis on exosomes as endogenous NPs capable of crossing BBs, which could be harbingers of a new era in medicine with their potential in numerous applications including prevention, intervention, and different types of drug and gene therapies. While synthetic NPs have seen limited clinical translation, exosomes may prove to supersede obstacles in delivering therapeutic substances with specificity, greater bioavailability, and reduced risks. Increasing information is shedding light on the function of EVs, with goals to manipulate these carriers to become specialized drug delivery systems (DDSs). However, the delivery mechanisms, cargo, composition, specificity, and uptake for crossing BBs are still not well understood and need further study. Some reported observations stated that exosomes can cross BBs in both directions, such as exosomes shedding from CNS neurons and glia being found in peripheral blood and in tears [245] due to the increased permeability of the BBB from neurodegenerative conditions. Exosome uptake can significantly alter the transcriptomic profile and dysregulate genes associated with tight junctions, resulting in a significant increase of cell membrane permeability and in a decrease of transendothelial electrical resistance [246]. Potential mechanisms for crossing the BBs are: (A) receptor-mediated transcytosis; (B) adsorptive transcytosis; (C) efflux; (D) carrier-mediated transport; (E) paracellular transport; (F) and diffusion [247]. The current most acceptable mechanism for explaining the exosome mechanism used to cross BBs is called the transcytosis mechanism, which is illustrated in Figure 2. In BBs, endothelial cells are polarized and bound by tight junctions, forming a seal that controls free movement or molecules from blood to tissue subendothelial space. Once an exosome is internalized, the intracellular pathway begins with the initial sorting as an early endosome. In polarized endothelial cells, endocytosis occurs at the apical and basolateral membranes with both processes generating their own early endosomes. The route back to the plasma membrane can occur directly from recycling endosomes, which is considered to be the major pathway for exosomes crossing BBs, either receptor-mediated transcytosis or adsorptive transcytosis [39,248,249].

Many receptors could mediate exosomes crossing BBs depending on exosome subtypes and sources of exosomes, which are still not well understood [250]. Thus, compiling data in a central source notating origin, innate targeting, recipient cells, and pathways is essential and could provide easier accessibility and cross-referencing for drug delivery and therapeutic development. This could shed new light on exosome roles and function within the body as a whole with centralized knowledge showing potential patterns. Caution is also imperative as they play significant roles in pathogenesis as well. However, exploring information in an organized, “one-glimpse” fashion could also allow researchers to utilize “pre-programmed” engineered exosomes for maximum uptake and potentially provide needed understanding of targeting specificity in the development of unique DDSs to overcome BBs, as well as other biological defense systems found within the body. Although ExoCarta and Vesiclepedia databases both already gathered a comprehensive list of exosomal molecular components, including proteins, nucleic acids, and lipids, the therapeutic function in terms of the specificity of exosomal markers to tissue targeting and ability across BBs would also be beneficial in such a central database. Compiled data in regard to exosome roles in normal as well as abnormal functions within the body could effectively increase understanding and help usher in a new era of targeted therapeutic options for intractable disorders and diseases, rapid diagnoses, and individualized medicine.

## Data Availability

Not applicable.
